# Immunohistochemical Expression of Programmed Cell Death Ligand 1 (PD-L1) in Human Cutaneous Malignant Melanoma: A Narrative Review with Historical Perspectives

**DOI:** 10.3390/genes14061252

**Published:** 2023-06-12

**Authors:** Gerardo Cazzato, Teresa Lettini, Anna Colagrande, Irma Trilli, Francesca Ambrogio, Carmelo Laface, Paola Parente, Eugenio Maiorano, Giuseppe Ingravallo

**Affiliations:** 1 Section of Molecular Pathology, Department of Precision and Regenerative Medicine and Ionian Area (DiMePRe-J), University of Bari, 70124 Bari, Italy; teresa.lettini@uniba.it (T.L.); anna.colagrande@gmail.com (A.C.); eugenio.maiorano@uniba.it (E.M.); giuseppe.ingravallo@uniba.it (G.I.); 2 Odontomatostologic Clinic, Department of Innovative Technologies in Medicine and Dentistry, University of Chieti, 66100 Chieti, Italy; irmatrilli@hotmail.com; 3 Section of Dermatology and Venereology, Department of Precision and Regenerative Medicine and Ionian Area (DiMePRe-J), University of Bari, 70124 Bari, Italy; dottambrogiofrancesca@gmail.com; 4 Medical Oncology, Dario Camberlingo Hospital, 72021 Francavilla Fontana, BR, Italy; carmelo.laface@gmail.com; 5 Pathology Unit, Fondazione IRCCS Ospedale Casa Sollievo della Sofferenza, 71013 San Giovanni Rotondo, FG, Italy; paolaparente77@gmail.com

**Keywords:** programmed death-ligand 1, PD-L1, anti-PD-1, nivolumab, pembrolizumab, immunotherapy

## Abstract

Programmed death-ligand 1 (PD-L1) is the primary ligand of the receptor programmed death-1 (PD-1) which is constitutively expressed or activated in myeloid, lymphoid (T, B and NK), normal epithelial cells, and cancer. The PD-1/PD-L1 interaction is crucial for the physiological development of immunological tolerance but also in the development of the cancer. Among these, malignant melanoma represents a tumour in which the immunohistochemical expression of PD-L1 is important to guide future therapeutic choices based on the presence/absence of expression. Various clones have been used over time for immunohistochemical determination, and different results and heterogeneity remain among the various studies in the literature. We perform a narrative review of the present studies in order to discuss and take stock of what certain achievements have been made in this field, what challenges remain, and what possible solutions can be found.

## 1. Introduction

Programmed death-ligand 1 (PD-L1) is the primary ligand of the receptor programmed death-1 (PD-1), which is constitutively expressed or activated in myeloid, lymphoid (T, B and NK), normal epithelial cells, and cancer [1].

The PD-1/PD-L1 interaction is crucial for the physiological development of immunological tolerance, which involves monitoring immune system over-reactions that could result in tissue destruction and/or the development of autoimmune diseases, potentially resulting in serious consequences [2]. PD-L1 expression can therefore be either constitutive or inducible [3]. In the first scenario, PD-L1 is expressed at a certain level on resting lymphocytes, antigen-presenting cells (APCs), as well as in corneal, syncytiotrophoblastic, and Langerhans cells where it contributes to promoting an intratissue balance that orchestrates and regulates inflammatory responses [2,3]. As a result, PD-L1 is able to confer a “privileged immune” status on some tissues, including the placenta, testis, and anterior chamber of the eye, allowing for the tolerization of external antigens without eliciting an inflammatory immune response [4]. On the other hand, haematopoietic, endothelial, and epithelial cells are stimulated to produce PD-L1 as a suppression signal during an infectious or inflammatory phase [4,5]. Thus, PD-L1 plays a role in the processes of central and peripheral tolerance, which are the negative selection of self-reactive lymphocytes in primary (central tolerance) and secondary (peripheral tolerance) lymphatic organs, respectively. It also plays a role in the so-called “immune exhaustion” process, which is the progressive decline of effector T-cell function after persistent antigen presentation, a physiological mechanism that prevents tissue destruction in chronic infections, and finally, it is also used by cancer cells to suppress the immune system’s activity in the regulation of the antitumour immune response [6]. Due to the latter feature, some cancer types use PD-L1 expression as a strategy to elude immune system cells, which may be related to a worse prognosis. The expression of PD-L1 on tumour cells assessed by immunohistochemistry (IHC) was initially identified as a biomarker for predicting response to treatment with anti-PD-1/anti-PDL1 therapies, and this topic has been extensively studied on different tumour types with conflicting results. Malignant melanoma represents one of the most “immunogenic” neoplasms in existence and this has allowed the testing, development, and application of immunotherapeutic drugs specifically in metastatic melanoma patients. In this narrative review, we address the issues of the immunohistochemical determination of PD-L1 in melanoma specimens, starting with early work that identified possible useful information and ending with the latest findings. We particularly focus on the use of the various PD-L1 clones commercially available and used in the studies discussed, with special attention to the latest current guidelines on the interpretation of PD-L1 in IHC.

In this narrative review, we extensively searched Medline, Scopus, and Pubmed for manuscripts describing the use of immunohistochemistry in the determination of PD-L1 expression, addressed issues related to the use of different clones, and also presented immunohistochemistry data in relation to different oncological outcomes, such as progression-free survival (PFS) and overall survival (OS) from January 2006 to January 2023. In particular, “PD-L1” or “Programmed Cell Death Ligand 1” and “malignant melanoma” or “melanoma” and “immunohistochemistry” and/or “IHC” were searched as keywords. We analysed any type of article without any restrictions, except that it had to be written in English.

## 2. History of PD-L1 in Malignant Melanoma

One of the earliest papers in literature concerning the immunohistochemical expression of PD-L1 was by Yang W. et al. [7], in which the authors evaluated the expression of this protein in nine primary and five metastatic uveal melanomas (UM). In addition to the use of cell lines, flow cytometry, and reverse-transcription polymerase chain reaction (RT-PCR), the authors used the MIH1 e-Bioscience clone of the monoclonal anti PD-L1 antibody. In all the cases they analysed, no PD-L1 expression was detected in situ, but together with the data from the cell lines and RT-PCR, the authors hypothesised that T-cells, by processing IFN-γ at the level of UM liver metastases, promoted the initiation of PD-L1 expression, which in turn reduced T-cell proliferation by hindering the production of interleukin-2 (IL-2). Some time later, Krönig H. et al. [8] conducted a study on the expression of PD-1 and its interaction with PD-L1 using 100 peripheral blood samples from stage I-IV MM patients and simultaneously performed immunohistochemical reactions on 37 primary/metastatic melanoma samples, testing Melan-A, PD-L1 and PD-1. All IHC investigations for PD-L1 were performed with polyclonal rabbit antibody ProSci, Poway, CA, USA) and expression was quantified at 400× magnification. In the case of primary melanomas, PD-L1 was analysed at the edges of the invasive front where the signal appeared most intense. Combining data from the peripheral blood mononuclear cells of HLA-A2+ patients and immunohistochemical results, the authors found that, compared to the entire CD8+ T-cell population, PD-1 expression from A2/Melan-A + CD8+ T cells was over-represented in stages III and IV, but although Melan-A + PD1 + T cells were elevated, this did not impact OS, while a positive correlation between PD-1 expression on MM cells and longer OS was described. The data reported by these authors were contrasted with a study by Hino et al. [9] in which it was reported that a high PD-L1 expression was an independent prognostic factor for a worse prognosis, although the clone used in the study by Hino was different (clone 27A2; MBL; Nagoya, Japan). On the other hand, the study by Gadiot et al. [10] also seemed to go in the same direction as Krönig.

In 2015, an interesting paper by Berghoff A.S. et al. [11] addressed the issue of PD-1 and PD-L1 immunoexpression in a sample of MM metastatic to the brain. Among other markers analysed (such as CD3+, CD8+, and CD45 RO), the authors reported PD-L1 expression in 22 samples of the 43 analysed and in 9/22 cases, PD-L1 was observed in >5% of the neoplastic cells. Furthermore, PD-L1 expression was associated with a higher density of PD-1, CD3, and FoxP3 TILs infiltration and these data were the basis for suggesting an implementation of therapeutic regimes in patients with brain metastases from MM.

A paper by Madore J. et al. [12] reported an interesting analysis of 139 samples from 58 MM patients (43 primary melanomas and 96 metastatic melanomas) and, when possible, studied individual patient samples longitudinally at various stages of disease, i.e., primary melanoma (PM), loco-regional metastases (LR), and distant metastases (DM). All analysed samples demonstrated a significant heterogeneity for PD-L1 expression both intra- and interpatient, using the cutoff >/=1% for PD-L1 positivity/negativity. Interestingly, when comparing longitudinal samples from each individual patient, there was no significant intrapatient concordance between PD-L1 status in the primary lesion and loco-regional malignancy, nor between a primary melanoma and distant metastases. Finally, there was no concordance of PD-L1 status in LR and DM. This paper clearly demonstrated that it was preferable, if possible, (1) to determine PD-L1 on excisional biopsies instead of incisional/punch biopsies; (2) to use the patient’s most recent metastatic specimen as PM was not a reliable source of predictive PD-L1 immunoexpression status in metastases; (3) to keep in mind the possibility of patients with atypical/outlier PD-L1 expression as they are candidates for a further molecular analysis to elucidate any mechanisms involved. In 2014, Massi D. et al. [13] reported on 81 consecutive cases of MM whose PD-L1 expression was studied using the antibody ab58810 polyclonal (Abcam, Cambridge, United Kingdom) and 5H1 monoclonal. The concordance data were very high between the two antibodies used and, in total, 40.3% of the metastatic melanoma samples were positive for PD-L1, compared to 14% of the primary melanomas. Using cell lines and pRT-PCR, it was shown that PD-L1 expression was an independent negative prognostic factor.

The paper by Kakavand H. et al. [14] was very interesting, in which the authors studied 93 tumours from 40 patients treated with a BRAF inhibitor (BRAFi, *n* = 28) or a combination of BRAF and MEKi (Combi, *n* = 12) whose samples were excised before treatment, early during treatment, and at progression. In addition to IHC staining for CD4, CD8, CD-68, LAG-3, and PD-1, staining for PD-L1 was performed with Merck’s monoclonal antibody 22C3, at a 1:1000 dilution. The authors noted that the lesions of patients who were positive for PD-L1 at baseline showed a significant decrease at progression, whereas the opposite was true (lesions negative for PD-L1 at baseline showed a significant increase at progression), regardless of treatment with BRAFi or Combi. Overall, PD-L1 expression was highly correlated with the presence of TILs. In another paper by Kakavand H. et al. [15], the authors tried to understand whether PD-L1 expression in MM cells present in the sentinel lymph node of positive patients could have any impact on their management and potential use of PD-1/PD-L1 inhibitors in the adjuvant setting. The metastasis-positive sentinel lymph nodes of 60 treatment-naive patients were analysed and, in addition to CD3, CD4, CD8, FoxP3, and PD-1, Merck’s monoclonal antibody 22C3, at a 1:1000 dilution, was used. Although the tumour expression of PD-L1 was present in 26/60 cases and did not correlate with outcomes, the authors showed that there was a positive correlation between recurrence-free/overall survival and the number of CD3+, CD4+, and CD8+, along with a negative correlation with the number of PD1+ T-lymphocytes in peritumoural distribution. It was very interesting to note that a certain microenvironment in the LN could predict the patients’ outcome.

Sunshine J.C. et al. [16] analysed very clearly the diagnostic performance of five different antibodies used in studies conducted on 34 MM samples from as many patients and broken down as 7 primary melanomas, 1 loco-regional localisation, and 27 metastatic melanomas. They used the clones 5H1, SP142, 28-8 (Dako PharmDx, Santa Clara, CA, United States), 22C3, and SP263, and the results showed a strong correlation between the staining patterns and the use of the different clones. When differences occurred, the spatial heterogeneity of the melanoma tissue section was responsible for the discrepancies rather than the variations in the PD-L1 antibody staining features. A strong correlation between PD-L1 intensity/H-scores and the proportion of PD-L1(+) cells was also found, and their findings further contradicted the idea that chromogenic PD-L1 IHC tests should include an intensity/H-score.

Kaunitz J.G. et al. [17] conducted an important study on the expression of immunohistochemistry for PD-L1 on 200 formalin-fixed paraffin-embedded (FFPE) specimens from patients with acral (*n* = 16), mucosal (*n* = 36), uveal (*n* = 103), and chronic sun-damaged (CSD) (*n* = 45) melanomas (24 lentigo maligna, 13 “mixed” desmoplastic, and 8 “pure” desmoplastic melanomas) in order to understand whether there was a differential expression of PD-L1 and CD8+ according to different MM histotypes.

The extent of the presence of CD8+ TILs was classified as mild, moderate, or severe, and the association between PD-L1 expression and location was examined, and in 31% of acral melanomas, 44% of mucosal melanomas, 10% of uveal melanomas, and 62% of CSD melanomas, PD-L1 expression was found. The proportion of PD-L1(+) lesions was lower in uveal and greater in CSD melanomas, although PD-L1 expression in the acral and mucosal subtypes was comparable to a precedent paper by the same authors. All subtypes of PD-L1 expression showed a moderate-to-severe CD8+ TIL grade correlation, supporting an adaptive mechanism of expression. The pure desmoplastic subtype of CSD melanomas, which expressed PD-L1 at lower levels than other subtypes, was associated with distinct tumour microenvironments. PD-L1 expression was not correlated with the existence of lymphoid aggregates; however, it was significantly higher in PD-L1(+) cases with spindle-cell shape than in patients with a nested phenotype in those cases.

In this field, numerous attempts have been made to develop standardised immunostaining protocols in order to succeed as much as possible in reducing the differences between the different assays [18,19,20,21,22].

In a paper by Ren M et al. [20], the analysis of 78 primary acral melanoma samples made it possible to study the expression characteristics of PD-L1 by correlating them with clinicopathological and survival parameters. The authors demonstrated that the expression of PD-L1 occurred at the tumour–stroma interface in tumour cells and TILs, which was consistent with the main pattern of TIL distribution. A high PD-L1 expression in cancer cells was also linked to the presence of peritumoral TILs. Furthermore, there was a strong correlation between the expression of PD-L1 in TILs. However, there was a lack of association among clinicopathological features and either PD-L1 expression in cancer cells or that in TILs. Cases with PD-L1-positive TILs showed a significantly worse survival in a univariate analysis than those with PD-L1-negative TILs. While PD-L1 expression in cancer cells was not substantially connected with survival in a univariate analysis or a multivariate analysis (*p* = 0.354), it was an independent predictor for poor prognosis in a multivariate analysis for TILs.

In a study by Koelblinger P. et al. [23], researchers investigated immunological variations in innate and adaptive immunity between ulcerated and nonulcerated primary melanomas and compared them to clinical outcomes.

The authors analysed 112 MM samples by studying various markers identifying TILs, macrophages, and dendritic cells and, in addition, analysed PD-L1 expression. Recurrence occurred in 21/56 patients (37.5%) with ulcerated tumours compared to 14/56 patients (25.0%) with nonulcerated tumours, and tumour ulceration was associated with a more frequent development of brain metastases (17.6 vs. 3.6% of patients). Immunohistochemistry showed an association of ulceration with the presence of intratumoural CD68+ macrophages and increased numbers of intratumoural CD11c+ dendritic cells and CD163+ macrophages. PD-L1 positivity (expression in >1% of tumour cells) was more frequent in ulcerated tumours than in nonulcerated tumours, and a positive correlation was found between the number of intratumoural CD11c+ and CD163+ cells and the frequency of PD-L1 expression of tumour cells.

These results confirmed the unfavourable clinical outcome associated with primary melanoma ulceration, particularly with regard to the risk of recurrence and the subsequent development of brain metastases. The immunological differences observed suggested a conceivable role of increased numbers of intratumoural macrophages and dendritic cells, associated with increased PD-L1 expression by tumour cells, which could contribute to the immunosuppressive and growth-promoting microenvironment of ulcerated primary melanomas.

Of great interest was the relationship found between CD8+ TIL expression and PD-L1 in a cohort of patients studied by Skuciova V. et al. [24], in which immunohistochemistry for PD-L1 and CD8 was performed on 56 formalin-fixed paraffin-embedded samples from patients with cutaneous and metastatic malignant melanomas using clone 28-8 and then, tumour proportion scores (TPS) were assessed. PD-L1 expression was detected in 20 of the 56 cases analysed, and PD-L1 expression on tumour cells was significantly increased with an increased infiltration of TIL into the tumour microenvironment; in addition, a lower TIL score corresponded to poorer prognostic clinicopathological parameters, such as a higher number of mitotic figures, Clark level and Breslow thickness.

Giavina-Bianchi M. et al. [25] highlighted a very important concept concerning PD-L1 immunoexpression in the metastatic setting by studying 50 melanoma metastasis samples from 46 patients on which they performed immunohistochemical reactions with the E1L3N (R) clone (Cell Signaling Technology, Danvers, Massachusetts, United States). The results showed that 22 patients expressed PD-L1 between 5% and 60%, with no statistically significant differences in gender, age, and phototype. Furthermore, the authors found that survival was not associated with PD-L1 expression even though they confirmed the results of their own previous study reporting PD-L1 positivity in nodular melanomas. The concept that was most emphasised was the extreme heterogeneity of PD-L1 protein expression in the metastatic setting, with the possibility that different metastatic organs could express the protein in totally different ways. This basis was necessary in order to speculate on the possibility that this extreme heterogeneity of immunoexpression could be the reason why metastatic patients responded to PD-1/PD-L1 inhibitors despite the absence of expression in a given sample.

In a recent paper from 2022, Yoneta D. et al. [26] addressed the issue of using different clones for the determination of PD-L1 immunoexpression. In detail, the authors analysed 64 samples consisting of 56 primary lesions and 8 metastatic lymph nodes from a total of 56 patients, distributed as follows: 28 acral melanomas, 8 mucosal melanomas, 28 cutaneous melanomas, and 2 unknown melanomas. The clones used were E1L3N, SP142, and 28-8 with a positivity cutoff of >/=1%. The positivity rates were 25.0% for 28-8, 34.0% for E1L3N, and 34.0% for SP142 in 64 samples. Acral melanoma positivity rates were 10.7% for 28-8, 21.4% for E1L3N, and 21.4% for SP142. The mucosal melanoma positivity rate for which all three antibodies reacted was 12.5%. The cutaneous melanoma positivity rates were 55.6% for 28-8, 66.7% for E1L3N, and 66.7% for SP142. Significant relationships were observed between PD-L1+ tumour cells, CD4+ TILs, and CD8+ TILs. The authors concluded that the results of staining with E1L3N, SP142, and 28-8 antibodies were within the permitted range, although the positivity rates of E1L3N and P142 were slightly higher than those of 28-8. CD4+ TILs and CD8+ TILs were quantitatively related to PD-L1-positive tumour cells.

## 3. PD-L1, TMB and Molecular Features

In addition to the use of PD-L1 in IHC, an important question was whether there could be other tools that could serve as biomarkers to predict the response of patients with metastatic cutaneous melanoma treated with immunocheckpoint inhibitors (ICI). With this in mind, Morrison C. et al. [27] conducted a multicentre study on samples of 300 patients who met certain criteria, including a history of metastatic cutaneous melanoma with previous excision of a primary/metastatic neoplasm; the availability of FFPE tissue prior to initiation of ICIs therapy; and the availability of sequencing data as well as demographic, diagnostic, follow-up, and vital status data. The IHC determination of PD-L1 was performed with the Dako Omnis platform (Agilent, Santa Clara, CA, USA) and 28-8 PharmDx antibody, and, in addition, the distribution of CD8+ T-lymphocytes was determined using anti-CD8 C8/144B antibody (Agilent, Santa Clara, CA, USA). The determination of mutational load and RNA-seq profiling were performed by the targeted capture and sequencing of 409 cancer-related genes and the amplicon sequencing of 394 immune transcripts on samples that were recognised to meet quality control thresholds. The authors described positivity for PD-L1 (>1% of membrane-tagged tumour cells) in 33% of cases (98/298 cases) and among ICI-treated patients, PD-L1 positivity was associated with a 55.6% objective response rate (ORR), while 37.9% of PD-L1- melanoma patients had an ORR after ICI. Further results showed that PD-L1 positivity on OS was much more considerable among patients undergoing ICI compared to the cohort of patients treated with conventional therapy (pre-ICI) that constituted the historical control group, confirming the predictive value of PD-L1 immunolabelling in patient stratification with a cutoff of 1%. In addition to the use of PD-L1 IHC and tumour mutational burden (TMB), the authors analysed the algorithmic response score (RS) and assessed how this parameter, being a continuous variable, could better serve as a decision support for the clinician; in fact, immune profiling with RS showed a higher sensitivity (72.2%) than PD-L1 IHC and TMB (32.5%) albeit with a similar specificity.

Therefore, this tool proved to be a valid method for predicting response to ICIs in patients with cutaneous melanoma metastases.

In a paper by Cho A.Y. et al. [28], the authors studied the correlation between PD-L1 expression and TMB values and microsatellite instability (MSI) in a cohort of 588 FFPEs from different types of malignancy, including melanoma samples. The TMB analysis of the melanoma samples (14 cases in total) revealed lower mutation number/Mb (megabase) values than data presented in other studies, and this was probably due to the fact that the melanoma sample analysed consisted more of the acral lentiginous histotype and the mucosal and uveal type.

The paper by Huan R.S.P. et al. [29] is very interesting, in which the authors analysed 1268 melanoma samples for which an IHC investigation for PD-L1 was requested and which were divided into high-pigmented melanoma (HPMel) and low-pigmented melanoma (LPMel), constituting 13.0% and 87.0% of the sample, respectively. In terms of TMB, in the HPMel cohort, TMB was significantly lower (median 8.8 mutations/Mb) than in the LPMel group (11.4 mutations/Mb), while secondary molecular alterations such as TERTp, CDKN2A, TP53, and PTEN were lower in HPMel cases than in LPMel ones. In contrast, a higher rate of genomic alterations in CTNNB1, APC, PRKAR1A, and KIT was identified in the HPMel cohort compared to the LPMel one. This paper had the merit of addressing and discussing how the high presence of melanin could be a limitation when performing immunohistochemical techniques for PD-L1 and that, in cases such as these, molecular approaches were more suitable, which seemed to indicate that in the case of HPMel, there were a lower TMB and molecular alterations than in LPMel cases.

In a paper by Shiu M.I. et al. [30], the authors retrospectively analysed 124 patients who had had at least one treatment with anti-PD1 monotherapy for metastatic acral or mucosal melanoma. Samples from these patients were collected at baseline and after treatment, in cases of resistant neoplasms, using IHC for PD-L1, whole-exome sequencing, and RNA sequencing. The authors reported that, at baseline, lesions that did not become resistant showed a higher PD-L1 expression, immune cell infiltration, and a high TMB; moreover, PD-L1 expression was also more common in secondary-resistant tumours than in primary-resistant tumours, and in late secondary-resistant tumours than in early resistant tumours. It was also interesting to note that melanomas that did not progress had higher 18-gene T-cell gene expression (T-cell inf GEP) and that, in treated cases, the GEP mRNA signature was increased, while the expression of the WNT and INFA1 pathway mRNA signatures showed a decrease. Last, but equally important, was the finding that there were changes in CD11c+ cell density at the beginning and compared to secondary-resistant tumours at the end.

## 4. Conclusions

From what has been said and addressed, it is clear how much scientific research is happening in an attempt to ensure the best reliable way to determine the extent of PD-L1 immunoexpression in melanoma. Various studies have looked at the different performance of commercially available clones, evaluating their efficacy and differences and concluding that the ranges of variability are quite acceptable. Furthermore, it would appear that some MM histotypes, such as nodular, are more likely to express PD-L1 than other histotypes, which have not yet been fully studied in this respect. It is also very important to emphasise the value of the cutoff chosen in the various studies, as it can impact and influence the data then reported by the pathologist to the clinician. Finally, it is important to emphasise how the heterogeneity of intra- and interpatient PD-L1 expression is very important when, in the metastatic setting, the decision of an anti-PD1 monotherapy or a combination is entrusted depending on the positivity/negativity of the protein. Therefore, it is crucial to take all these multiple aspects into account when dealing with this topic, remembering that the translational potential of this immunohistochemical determination is very high.

## Data Availability

Not applicable.
